# Gender-Specific Hippocampal Dysrhythmia and Aberrant Hippocampal and Cortical Excitability in the APPswePS1dE9 Model of Alzheimer's Disease

**DOI:** 10.1155/2016/7167358

**Published:** 2016-10-20

**Authors:** Anna Papazoglou, Julien Soos, Andreas Lundt, Carola Wormuth, Varun Raj Ginde, Ralf Müller, Christina Henseler, Karl Broich, Kan Xie, Dan Ehninger, Britta Haenisch, Marco Weiergräber

**Affiliations:** ^1^Department of Neuropsychopharmacology, Federal Institute for Drugs and Medical Devices (Bundesinstitut für Arzneimittel und Medizinprodukte (BfArM)), Bonn, Germany; ^2^German Center for Neurodegenerative Diseases (Deutsches Zentrum für Neurodegenerative Erkrankungen (DZNE)), Bonn, Germany; ^3^Department of Psychiatry and Psychotherapy, University of Cologne, Cologne, Germany

## Abstract

Alzheimer's disease (AD) is a multifactorial disorder leading to progressive memory loss and eventually death. In this study an APPswePS1dE9 AD mouse model has been analyzed using implantable video-EEG radiotelemetry to perform long-term EEG recordings from the primary motor cortex M1 and the hippocampal CA1 region in both genders. Besides motor activity, EEG recordings were analyzed for electroencephalographic seizure activity and frequency characteristics using a Fast Fourier Transformation (FFT) based approach. Automatic seizure detection revealed severe electroencephalographic seizure activity in both M1 and CA1 deflection in APPswePS1dE9 mice with gender-specific characteristics. Frequency analysis of both surface and deep EEG recordings elicited complex age, gender, and activity dependent alterations in the theta and gamma range. Females displayed an antithetic decrease in theta (*θ*) and increase in gamma (*γ*) power at 18-19 weeks of age whereas related changes in males occurred earlier at 14 weeks of age. In females, theta (*θ*) and gamma (*γ*) power alterations predominated in the inactive state suggesting a reduction in atropine-sensitive type II theta in APPswePS1dE9 animals. Gender-specific central dysrhythmia and network alterations in APPswePS1dE9 point to a functional role in behavioral and cognitive deficits and might serve as early biomarkers for AD in the future.

## 1. Introduction

Alzheimer's disease (AD) is a multifactorial disorder leading to progressive memory loss and eventually death. One of the histopathological hallmarks of AD is the excessive accumulation of amyloid beta (A*β*) peptides in the brain resulting in extracellular amyloid plaques [1–3]. Amyloid peptides are natural cleavage products derived from the amyloid precursor protein (APP) via sequential endoproteolytic processing operated by specific secretases, beta-site amyloid precursor protein cleaving enzyme 1 (BACE-1), and *γ*-secretase [4]. Length of A*β* peptides ranges from 36 to 43 amino acids [5]. While A*β*_1–40_ and A*β*_1–42_ represent the predominant isoform, abundance of A*β*_1–40_ is generally higher compared to A*β*_1–42_ which is prone to aggregate and exhibits stronger cytotoxicity [6]. APP mutations can be proamyloidogenic facilitating the generation of cytotoxic A*β* peptides. For instance, the Swedish double mutation KM670/671NL promotes BACE cleavage and increases abundance of both A*β*_1–40_ and A*β*_1–42_ whereas mutations at amino acid 717 augment generation of the more toxic A*β*_1–42_ [7]. Presenilin- (PS) 1 and presenilin-2 act as catalytic sites for *γ*-secretase and mutations in PS, which are much more common than APP mutations, further enhance the production of proamyloidogenic A*β*_1–42_ [8]. Besides the formation of A*β* plaques, human AD neurons also present neurofibrillary tangles, that is, intraneuronal inclusions of hyperphosphorylated tau (*τ*) protein [9, 10].

Numerous transgenic mouse models of AD exist that are mainly characterized by overexpression of one or more mutant APPs resulting in excessive amounts of A*β*_1–42_. The latter causes age-related AD-specific abnormalities including A*β* plaques, axonal and synaptic dystrophy, impaired synaptic plasticity, and impaired learning and memory [11–14]. In this study an APPswePS1dE AD mouse model has been used which is characterized by carrying human APP with Swedish double mutation (APPswe) cointegrated with human PS-1 with exon 9 deletion (PS1dE9) [15–17]. This leads to overproduction of APP and PS1 splice variants with subsequent increase in neural A*β* load.

About 100 AD mouse models have been established so far and they differ significantly in pathohistomorphology, symptomatology, disease progression, mortality, and translational categories such as isomorphism, homology, and predictability [18]. In general, transgenic AD lines display A*β*_1–42_ overload and this might be related to increase in mortality and death with the latter occurring out of a sudden in most cases [19–21]. In the APPswePS1dE9 line the mortality was reported to be 10–15% irrespectively of pathomorphological findings [22]. APPswePS1dE9 mice develop first A*β* plaques around 4 months of age, particularly in the cortex and hippocampus. This coincides with a mortality peak around 3-4 months of age [23, 24]. At the age of 6 months memory deficits in radial arm water maze are prominent [25] whereas, at 12 months, mice start exhibiting behavioral and cognitive deficits detectable in spatial navigation, reference learning, and Morris water maze. It has been hypothesized that seizure activity might account for sudden death in this line as intracerebral A*β* accumulation was suggested to be causally linked to epileptogenesis [26, 27]. In addition, neurodegenerative processes in the cortex and septohippocampal system can result in complex central dysrhythmia [28] particularly affecting theta and gamma activity.

Previous studies have analyzed selective electrical activity and individual frequency characteristics from electrocorticograms and other deflections including sleep studies in APP transgenic mice [4, 22, 26, 29–31]. In contrast to these studies, we present a systematic FFT based frequency analysis and multiparameter longitudinal seizure evaluation from both cortical M1 and hippocampal CA1 recordings in APPswePS1dE9 and controls based on nonrestraining EEG radiotelemetry. Also for the first time we perform gender-specific analysis that reveals striking age-dependent differences in theta/gamma activity between male and female AD mice gaining further insight into gender-specific differences in the pathogenesis and symptomatology of AD.

## 2. Materials and Methods

### 2.1. Study Animals

This study was performed in APPswePS1dE9 transgenic mice with a C57BL/6J background carrying a human APP with Swedish double mutation (APPswe) cointegrated with human PS1 with exon 9 deletion (PS1dE) [15, 16]. Animals (B6.Cg-Tg(APPswe, PSEN1dE9)85Dbo/Mmjax, MMRRC stock no. 34832-JAX) were purchased from Jackson Laboratory. In total, 21 WT controls (10 males, body weight: 26.94 ± 0.64 g; 11 females, body weight: 21.23 ± 0.53 g) and 20 APPswePS1dE mice (9 males, body weight: 26.16 ± 0.56 g; 11 females, body weight: 21.73 ± 0.35 g) were analysed in this study separately. Body weight was determined at 14 wks of age. Importantly, both genders were used and analysed in this study. Due to the long-term experimental character and experimental time schedules (see the following), females could not be recorded within the same stage of the estrous cycle. In addition, the stages of the estrous cycle were reported not to influence basic EEG related parameters [31]. All experimental mice were housed in groups of 3-4 in clear Makrolon cages type II with ad libitum access to drinking water and standard food pellets. Mice were maintained at a temperature of 21 ± 2°C, 50–60% relative humidity, and on a conventional 12 h/12 h light/dark cycle beginning at 5:00 am using ventilated cabinets (Model 9AV125P). Subsequent epidural, that is, electrocorticographic and deep, intracerebral, that is, electrohippocampographic, recordings were performed inside the ventilated cabinets.

All animal experimentation was performed according to the Guidelines of the German Council on Animal Care and all protocols were approved by the Local Institutional and National Committee on Animal Care (Landesamt für Natur, Umwelt und Verbraucherschutz, LANUV, Germany). The authors further certify that all animal experimentation was carried out in accordance with the European Communities Council Directive of November 24, 1986 (86/609/EEC) and of September 22, 2010 (2010/63/EU). Special attention was paid to minimize the animal sample size and the suffering of mice.

### 2.2. EEG Radiofrequency Transmitter Implantation

Mice were anesthetized using the inhalation narcotic isoflurane (Baxter 100% V/V). The volatile anesthetic was applied via facemask using a Matrix TM VIP 3000 Calibrated Vaporizer and a scavenger system from Harvard apparatus (USA) as described previously [32–34]. The radiofrequency transmitter TL11M2-F20-EET (2-channel transmitter, Data Science International (DSI, Germany) (specifications: weight 3.9 g, volume 1.9 cc, input voltage range ±1.25 mV, amplification factor (voltage gain) 200, and nominal sampling rate 250 Hz) was implanted into a subcutaneous pouch on the back of the experimental animals. The EEG electrodes of both radiotelemetry transmitter channels were stereotaxically positioned via a computerized 3D stereotaxic StereoDrive system (Neurostar, Germany) [32–34].

### 2.3. Epidural Electrode Placement for Electrocorticographic Recordings

The differential epidural surface electrode of channel 1 of the TL11M2-F20-EET transmitter was positioned at the following stereotaxic coordinates referring to the Bregma craniometric landmark: (+)-lead, cranial +1 mm, and lateral of Bregma 1.5 mm (left hemisphere). The differential electrode targets the primary motor cortex (M1). An epidural reference electrode was placed on the cerebellar cortex at Bregma −6 mm, lateral of Bregma 1 mm (left hemisphere). Note that for epidural electrode placement the sensing lead of the transmitter is bent 90° and directly placed on the surface of the cortex.

### 2.4. Intrahippocampal Electrode Placement for Electrohippocampal Recordings

For deep, intracerebral EEG recordings targeting the hippocampal CA1 region, the differential electrode of channel 2 of the TL11M2-F20-EET transmitter was positioned as follows: (+)-lead, caudal −2 mm, lateral of Bregma 1.5 mm (right hemisphere), and dorsoventral (depth) 1.5 mm. An epidural reference electrode was placed on the cerebellar cortex at Bregma −6 mm and lateral of Bregma 1 mm (right hemisphere). For deep recordings, the sensing lead of the transmitter is mechanically attached to the deep electrode. The deep tungsten electrodes (FHC, Bowdoin, USA) are insulated with epoxylite with a shank diameter of 250 *μ*m and an impedance of 50–100 kΩ (measured at 1000 Hz). Surface and deep electrodes were fixed using glass ionomer cement (Kent Dental, UK) and the scalp was closed using over-and-over sutures (Ethilon, 6-0). As mice are predisposed to hypothermia, supplemental warmth was given to the animal with a heating pad during the whole surgical procedure. A detailed description of the stereotaxic electrode placement and transmitter implantation was previously described in detail by Weiergräber and colleagues [32–34]. Carprofen (5 mg/kg, Rimadyl, Parke-Davis/Pfizer, Germany) was administered subcutaneously for postoperative pain management. Animals were given 10 days after surgery to fully recover. This recovery period is based on the observation that no differences in basic physiological/behavioral parameters such as food and water uptake, motor activity, and body temperature could be detected between radiotransmitter implanted, nonimplanted, and sham-operated mice 10 days after surgery [35].

### 2.5. Confirmation of EEG Electrode Placement

To verify whether electrodes were placed in the correct target region, brains were extirpated postmortem and fixed in 4% paraformaldehyde. Subsequently, brains were cut to 60 *μ*m slices using a Vibroslice Tissue Cutter EMS 5000-MZ (Campden Instruments Limited, UK). Slices were hematoxylin-stained for visualization of the branch canal. Animals, in which electrodes turned out to be not in the exact target position, were excluded from analysis.

### 2.6. Radiotelemetric EEG Data Acquisition

The first long-term recording of 48 hrs was performed at day 10 after surgery from both the primary motor cortex (M1) and the CA1 hippocampal region. A second 48 hrs long-term recording was performed at day 17 after implantation using both deflections. For EEG data acquisition, the Dataquest ART 4.2 software (DSI) was used. No a priori filter cutoffs were applied. The nominal sampling rate (f) of the TL11M2-F20-EET transmitter is 250 Hz. The virtual interpolated sampling rate was manually adjusted in the Dataquest ART 4.2 configuration window to 500 Hz per channel for better visualisation. Note that analysis was performed up to 70 Hz, considering the transmitter specific bandwidth and the Nyquist-Shannon limit of 125 Hz for this transmitter type.

### 2.7. Telemetric Activity Recording and Analysis

As the animal moves about in its cage, the telemetry signal transmitted to the receiver antennas varies in strength. The signal strength may vary due to orientation of the animal relative to the receiver or due to the distance of the animal from the receiver antennas. When the signal strength changes by a certain amount, an activity count is generated. The number of counts generated is dependent on both distance and speed of movement (acceleration). Note that the activity data provided by Dataquest ART is a relative measure of locomotor activity. Activity analysis was carried out for controls and APPswePS1dE9 mice from both genders.

### 2.8. Electroencephalographic Seizure Analysis

For detailed analysis data were exported to NeuroScore 2.1 (DSI). Qualitative and quantitative seizure analysis was carried out using the NeuroScore seizure detection and quantification module. As the seizure detection module requests configuration of the detection tool, spike detection characteristics including absolute threshold, spike duration limits, and spike interval limits were adapted for different seizure protocols. Using the absolute threshold approach for ictal discharge detection, the maximum amplitude was set as 1000 *μ*V with an average spike detection threshold value of 200 *μ*V. In both seizure detection protocols the minimum spike duration was determined as 1 ms and the maximum spike and short/slow-wave duration at 100 ms. Spike trains were detected with a minimum train duration of 0.5 s including at least 4 individual spikes. Spike intervals within a train were ranging between 0.05 and 0.3 s. The interval between individual spike trains was determined at 1 s. Application of seizure protocols resulted in total number of seizure episodes and spikes, spike frequency, total spike train duration, and shortest and longest spike train duration. Data were presented as mean ± standard error of the mean (SEM). Significance was calculated using Student's *t*-test which included pretesting for normal distribution via the Kolmogorov-Smirnov test. All statistics and graph presentation in this section were performed with GraphPad Prism 6 for Windows.

### 2.9. Analysis of Electrocorticographic and Electrohippocampographic Data

Recordings (48 hrs) of spontaneous EEG activity were performed based on a nominal sampling rate of 250 Hz of the radiofrequency transmitter and a virtual (software based) sampling rate of 500 Hz (per channel). EEG data were FFT analyzed using NeuroScore 2.1 (DSI) in the frequency range of 0.5–70 Hz, thus comprising the typical delta (0.5–4 Hz), theta (4–8 Hz), beta (12–30 Hz), and gamma bands of 30–50 Hz and 50–70 Hz. The upper gamma limit (70 Hz) is still below the Nyquist-Shannon limit of 125 Hz; thus FFT based analysis is possible [36]. The length of the individual EEG epochs that were FFT analyzed was 2 s. Subsequently, mean EEG power [%] was calculated for the individual frequency ranges, for both genders and the individual circadian stages, that is, two dark (D1, D2) and two light cycles (L1, L2). In addition, activity data of mice during the conventional 12 h light/dark cycle (starting at 5:00 a.m.) were used to correlate activity in different EEG frequency bands from both deflections with either the active (activity units > 0) or inactive state (activity units = 0).

Data were statistically analyzed and displayed as mean ± SEM. Statistics for frequency analysis were carried out using multiple Student's* t*-test, corrected for multiple comparison using the Holm-Sidak method (^*∗*^ *p* < 0.05; ^*∗∗*^ *p* < 0.01; ^*∗∗∗*^ *p* < 0.001). Most of the statistics and graph presentations were performed with GraphPad Prism 6 for Windows. Additional statistics (ANOVA) were computed with IBM SPSS® 23.

## 3. Results

### 3.1. Experimental Animals: Controls and APPswePS1dE9

As some Alzheimer mouse lines were reported to exhibit developmental deficits, we closely monitored the gender-specific body weight development of APPswePS1dE9 and control mice. In age-matched transgenic and control mice it turned out that males exhibited significant higher body weight compared to females. However, different from other Alzheimer mouse models such as the 5XFAD [27, 28] no significant reduction of body weight in APPswePS1dE9 could be detected compared to controls in our study at 14 wks of age (control males 26.94 ± 0.64 g (*n* = 10) versus transgenic males 26.16 ± 0.56 g (*n* = 9), *p* = 0.3755; control females 21.23 ± 0.53 g (*n* = 11) versus transgenic females 21.73 ± 0.35 g (*n* = 11), *p* = 0.4416).

### 3.2. Qualitative Electroencephalographic Seizure Characteristics in Controls and APPswePS1dE9 Mice

**Figure 1:**
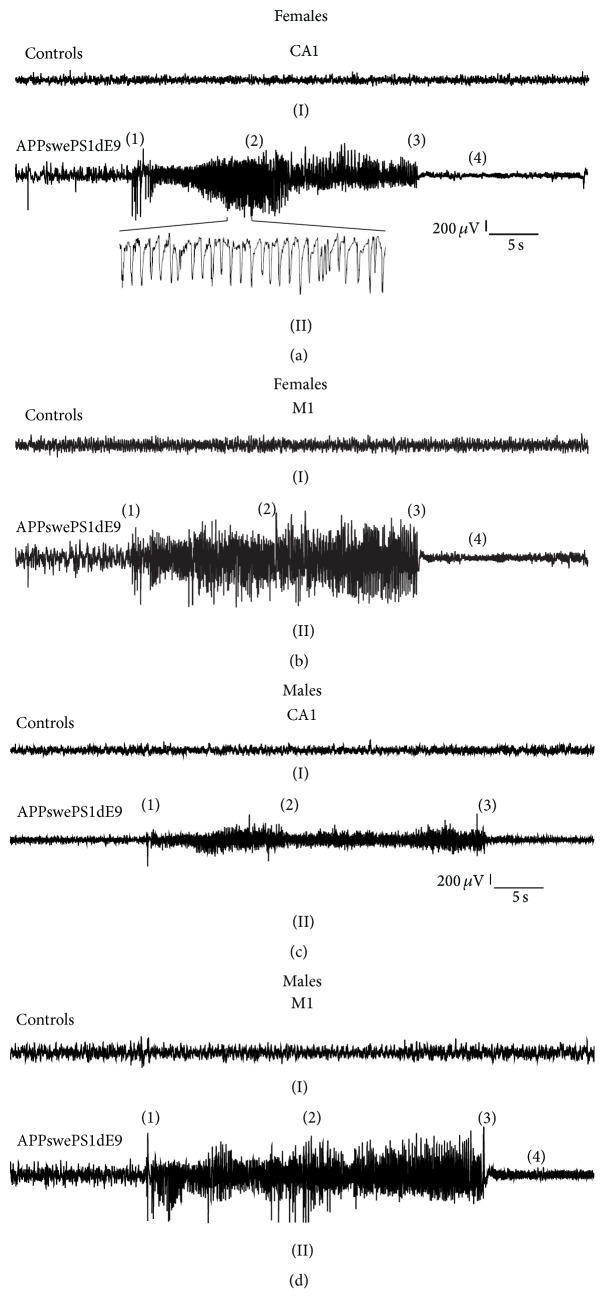
Ictal discharges (electroencephalographic seizure activity) in APPswePS1dE9 Alzheimer mice. (a and b) Representative 1 min EEG traces recorded from female controls (I) and APPswePS1dE9 mice (II) using a deep, intracerebral CA1 deflection (a) and an epidural surface electrode on the primary motor cortex (M1, b). Note that Alzheimer mice (II) display spike/spike train episodes which are characterized by spiking initiation (1), continuation (2), and termination (3). Reduced EEG amplitude following seizure termination represents postictal depression (4). Electroencephalographic seizure activity is present in both cortical and intrahippocampal recordings. (c and d) Representative 1 min EEG traces from male controls (I) and Alzheimer mice (II) obtained from CA1 (c) and M1 (d) deflections. As for females, spike/spike-wave train episodes are prominent in APPswePS1dE9 animals (time bar, 5 s; voltage bar, 200 *μ*V).

The 48 hrs CA1 and M1 long-term EEG recordings at the age of 14, 15, 18, and 19 weeks from both control mice and APPswePS1dE9 animals were visually analyzed for electroencephalographic seizure activity. As illustrated in Figures 1(a)–1(d), APPswePS1dE9 exhibited typical ictal like discharges, including spikes, spike-waves, and polyspike waves. Despite isolated ictal discharges we could also observe characteristic spike/spike-wave trains including the seizure initiation phase, seizure continuation, and termination (Figures 1(a)–1(d)). As reported previously [22], seizures often began with a large-amplitude spike and subsequent spike trains were typically, however not always followed by so-called postictal depression (Figures 1(a)–1(d)). Abnormal electroencephalographic ictal discharges were present in both CA1 and M1 deflections from both genders. In controls, ictal discharges were hardly detected and could be related to behavioral phenomena, for example, eating and grooming. In this study, we did not analyze motoric exacerbation of aberrant hyperexcitability, that is, behavioral seizure activity, but strictly focused on electroencephalographic seizure activity.

### 3.3. Motor Activity in Controls and APPswePS1dE9 Mice

**Figure 2:**
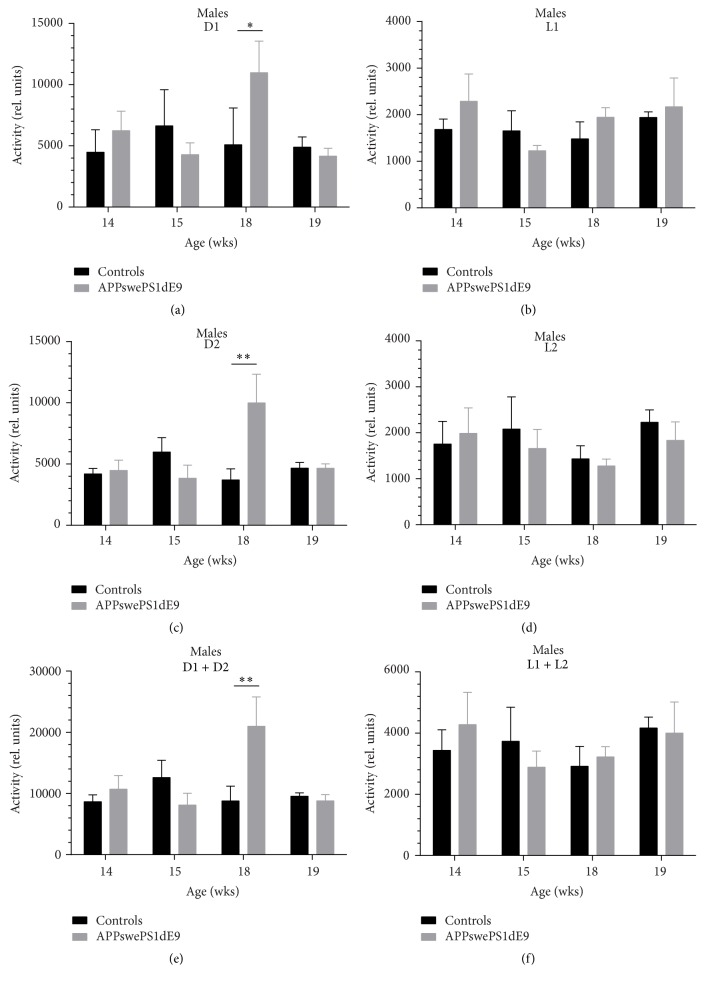
Activity analysis in controls and APPswePS1dE9 mice. Animals from both groups were analyzed for motor activity (relative units) for different ages (14, 15, 18, and 19 wks) and circadian periods (D1, L1, D2, L2, D1 + D2, and L1 + L2). Male Alzheimer mice exhibited significant increase in relative activity compared to controls at the age of 18 wks during the dark cyle(s) (D1, D2, and D1 + D1). In females, no changes were observed in motor activity (see Supplementary Figure  1). ^*∗*^ *p* < 0.05. ^*∗∗*^ *p* < 0.01.

We analyzed motor activity in controls and APPswePS1dE9 mice as both seizure activity and frequency characteristics can be associated or modified by motoric behavior (Figure 2). The activity was calculated for the first (D1) and second (D2) dark cycle (of each 48 hrs recording) and for the total dark period (D1 + D2). The same holds true for the two light cycles (L1, L2 of each 48 hrs recording) as well as the total light period (L1 + L2). In females, no significant difference in motor activity could be detected between controls and APPswePS1dE9 mice at any circadian stage (see Supplementary Figure  1 in Supplementary Material available online at http://dx.doi.org/10.1155/2016/7167358). In males, a significant increase in relative motor activity was observed during the dark cycle (D1, 5089.50 ± 1504.47 versus 10983.50 ± 2567.92, *p* = 0.0412; D2, 3700.25 ± 909.43 versus 9994.00 ± 2333.55, *p* = 0.0049; D1 + D2, 8789.75 ± 2410.34 versus 20977.50 ± 4812.86, *p* = 0.0127) at the age of 18 wks (Figures 2(a), 2(c), and 2(e)). However, no additional alteration in motor activity could be detected in males at any other time point.

### 3.4. Quantitative Seizure Analysis in Controls and APPswePS1dE9 Mice

**Figure 3:**
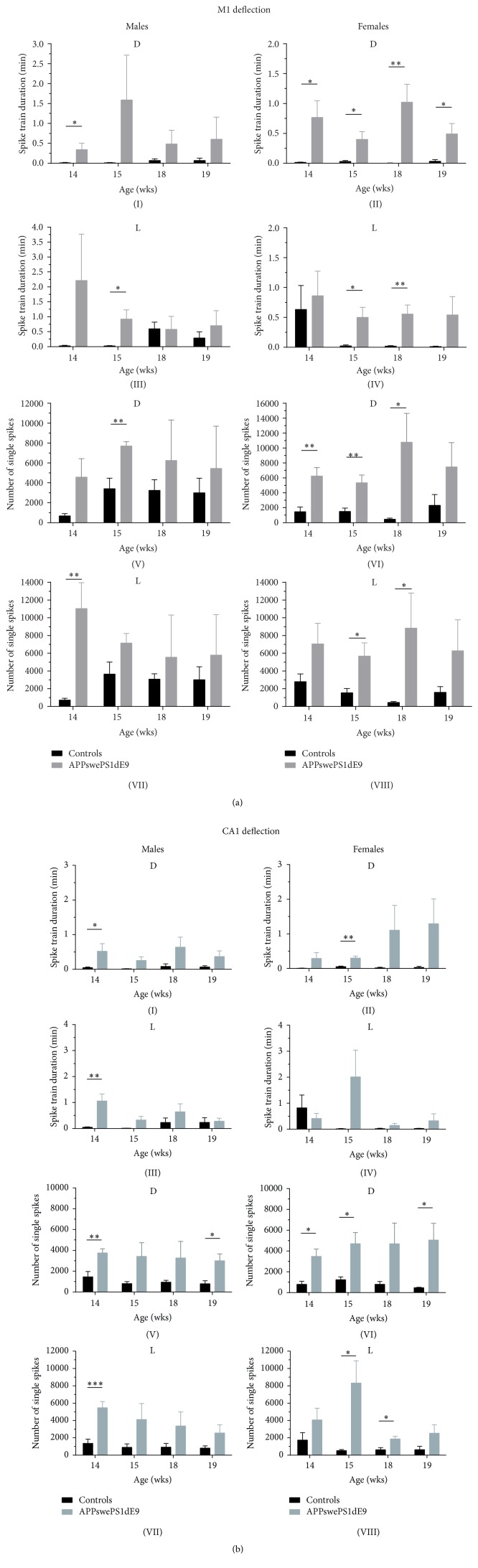
Electroencephalographic seizure analysis in controls and APPswePS1dE9. Both M1 (a) and CA1 (b) 48 hrs long-term EEG recordings were analyzed for electroencephalographic seizures using an automated seizure detection tool. Seizure parameters included the total spike train duration and the total number of spikes and were averaged for a single dark (D) or light (L) cycle for the various ages (14, 15, 18, and 19 wks). ^*∗*^ *p* < 0.05; ^*∗∗*^ *p* < 0.01; ^*∗∗∗*^ *p* < 0.001, level of significance.

In order to quantify electroencephalographic seizure activity in controls and APPswePS1dE9 mice we used an automatic seizure detection tool. This tool was applied for the individual circadian stages, that is, the dark (D) and light (L) stage, and various seizure parameters were evaluated, including the number of spike trains, total spike train duration, average spike train duration, and number of single spikes. These parameters were calculated again for both CA1 and M1 deflections in both genders. As it has been reported for other Alzheimer mouse models as well [26, 28], APPswePS1dE9 mice exhibit a high variability in seizure severity (Figure 3). Significant increases in spike/spike train parameters were detected consistently in M1 and CA1 deflections from both genders. Note that overall electroencephalographic seizure activity in males obtained from M1 and CA1 is most prominent at an age of 14-15 wks (Figures 3(a)(I, III, V, and VII) and 3(b)(I, III, V, and VII)) whereas related activity in females is also present at 18-19 wks of age (Figures 3(a)(II, IV, VI, and VIII), and 3(b)(II, IV, VI, and VIII); see also Supplementary Figure  2). This observation clearly correlates with proven age- and gender-specific differences in plaques formation in APPswePS1dE9 mice [37].

### 3.5. Frequency Analysis in Controls and APPswePS1dE9 Mice

**Figure 4:**
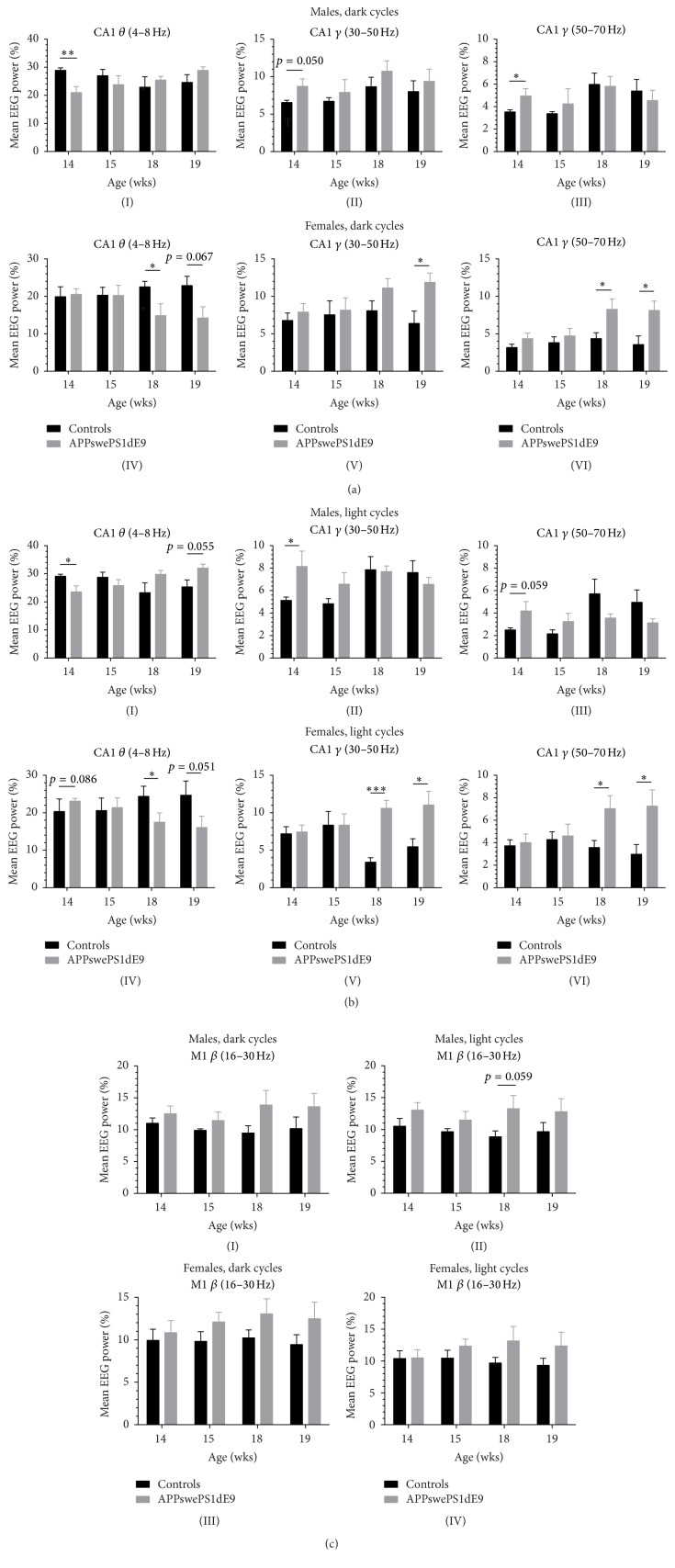
Frequency analysis in controls and APPswePS1dE9 mice. Using a FFT based approach, the mean EEG power [%] was calculated for males and females considering potential circadian rhythmicity (light/dark phase). For CA1 deflections, theta (4–8 Hz), gamma (30–50 Hz), and gamma (50–70 Hz) were quantified, for the cortical deflection delta (0.5–4 Hz) and beta (16–30 Hz). Frequency analysis was performed for all four ages (14, 15, 18, and 19 wks). ^*∗*^ *p* < 0.05. ^*∗∗*^ *p* < 0.01. ^*∗∗∗*^ *p* < 0.001.

In male APPswePS1dE9 mice a significant reduction in CA1 relative theta power was observed at the age of 14 wks (dark cycle: 28.890 ± 0.902 versus 20.978 ± 2.125, *p* = 0.0052; light cycle: 29.101 ± 0.754 versus 23.578 ± 2.091, *p* = 0.0253) but not later (Figures 4(a)(I) and 4(b)(I)). Interestingly, female APPswePS1dE9 mice also exhibited significant reduction in theta activity. However, this phenomenon occurred at later stage (18 wks, dark cycle: 22.528 ± 1.485 versus 14.870 ± 3.136, *p* = 0.0495; light cycle: 24.394 ± 1.201 versus 17.487 ± 2.484, *p* = 0.0313) and at 19 wks of age (dark cycle: 22.898 ± 2.458 versus 14.245 ± 3.002, *p* = 0.0673; light cycle: 24.691 ± 1.895 versus 16.084 ± 2.994, *p* = 0.0512) (Figures 4(a)(IV) and 4(b)(IV)). These findings clearly point to a circadian independent alteration in theta architecture and the developmental time-dependent characteristics of theta are gender-specific. In contrast to other Alzheimer mouse models that exhibit an increase in theta activity [28, 38], the APPswePS1dE9 model presents age-specific reduction in theta power that is in line with many other AD mouse lines [39–41].

Interestingly, the age-specific theta activity pattern in both genders is mirrored in the gamma activity in the CA1 deflection as well. Male APPswePS1dE9 mice exhibit an increase in low gamma (30–50 Hz) at early age of 14 wks (dark cycle: 6.556 ± 0.298 versus 8.713 ± 0.992, *p* = 0.0502; light cycle: 5.138 ± 0.296 versus 8.154 ± 1.367, *p* = 0.0424, Figures 4(a)(II) and 4(b)(II)) which is not observed in females. In contrast, females display significant increase in low gamma (30–50 Hz) at the age of 18 wks (dark cycle: 8.083 ± 1.314 versus 11.114 ± 1.250, *p* = 0.1455; light cycle: 3.406 ± 0.583 versus 10.611 ± 1.051, *p* < 0.001) and 19 wks (dark cycle: 6.399 ± 1.633 versus 11.886 ± 1.236, *p* = 0.0366; light cycle: 5.468 ± 1.065 versus 11.045 ± 1.836, *p* = 0.0391) (Figures 4(a)(V) and 4(b)(V)). The same pattern was observed for higher gamma frequency of 50–70 Hz. Male APPswePS1dE9 again showed increased activity at 14 wks of age (dark cycle: 3.540 ± 0.182 versus 4.956 ± 0.649, *p* = 0.0481; light cycle: 2.508 ± 0.189 versus 4.193 ± 0.834, *p* = 0.0593, Figures 4(a)(III) and 4(b)(III)), whereas females presented an increase at later stages, that is, 18 wks (dark cycle: 4.351 ± 0.787 versus 8.273 ± 1.379, *p* = 0.0351; light cycle: 3.572 ± 0.636 versus 7.038 ± 1.146, *p* = 0.0266) and 19 wks (dark cycle: 3.556 ± 1.159 versus 8.128 ± 1.245, *p* = 0.0362; light cycle: 2.981 ± 0.845 versus 7.265 ± 1.441, *p* = 0.0427) (Figures 4(a)(VI) and 4(b)(VI)). Note that analysis of higher gamma frequency is not recommendable/possible given the transmitter bandwidth of 1–50 Hz and the Nyquist-Shannon limit of the transmitter of 125 Hz. In summary, theta activity decrease and gamma activity increase are temporarily coupled in a gender-specific manner. We further tested this hypothesis with a univariate two-way ANOVA with between-subjects factors (i) genotype, that is, controls and APPswePS1dE9, and (ii) frequency band, that is, theta and gamma band power for CA1. Based on the previous statistical results, we choose data of 14-week-old male mice and 18-week-old female mice. For each gender, the subgamma band with the highest *p* value was selected. It turned out that this results in a cycle-dependent difference, but not a gender-dependent difference (dark cycle: higher gamma band (50–70 Hz) for both male and female; light cycle: lower gamma band (30–50 Hz) for both male and female). Based on this procedure, the statistical computations lead to a significant interaction between factor (i) genotype and factor (ii) frequency band for all four constellations (males, dark cycle: *p* = 0.0006; males, light cycle: *p* = 0.0083; females, dark cycle: *p* = 0.0058; females, light cycle: *p* = 0.0002), which demonstrates a clear connection between the decrease of theta power and the increase of gamma power. In addition to the gender-specific FFT based EEG power analysis we further checked for activity dependent power alterations between the active and inactive state (Supplementary Figure  3). During the dark cycle, it turned out that significant changes in males could not be detected. In females, the observed changes (Figure 4) persisted, particularly in the inactive state. This held true for the theta and both gamma frequency ranges analyzed. In the light phase, males displayed only minor changes in the theta range, but not the gamma ranges. Females, however, again exhibited prominent alterations in theta and both gamma ranges predominantly in the inactive stage. The antithetic distribution of reduced theta and increased gamma power in females was most outstanding during the inactive state (Supplementary Figure  3). We also performed FFT based EEG power analysis for M1 recordings from controls and APPswePS1dE9. No changes were observed for the beta frequency range (16–30 Hz, Figure 4(c)). For the delta range (0.5–4 Hz), there turned out to be only a significant reduction in relative power in males during light cycle at the age of 18 wks (30.469 ± 2.189 versus 18.426 ± 2.876, *p* = 0.0158).

## 4. Discussion

Alzheimer's disease is a neurodegenerative disorder that is accompanied by neural cell loss that ultimately results in neural network dysfunction, such as dysrhythmia and/or aberrant excitability. In humans, AD goes together with an increase of prevalence of spontaneous, unprovoked seizures in the elderly [42–45]. Within the last years there is increasing evidence that this phenomenon also holds true for various AD mouse models [22, 26–28, 31]. The mechanisms underlying ictogenesis in APP transgenic mice still remain poorly understood [26]. It is assumed that A*β* is a crucial trigger of ictogenesis/epileptogenesis in AD mice. This is further supported by the observation that exogenous administration of A*β*_1–42_ can lead to neural hyperexcitability in cortical and hippocampal slices of WT mice [22]. Whether A*β* plaques or high A*β* concentrations exhibit a higher proictogenic potential has not been strictly proven yet. Importantly, seizures tend to peak around an age when amyloid plaques occur in both the cortex and hippocampus [31]. Some previous studies have analyzed spontaneous electrical activity in electrocorticograms and carried out sleep studies in APP transgenic mice [4, 22, 23, 26, 29]. Unlike our nonrestraining EEG radiotelemetry approach, Minkeviciene et al. [22] performed EEG recordings in APPswePS1dE9 mice from the frontal cortex using a restraining tethered system. Whereas Minkeviciene et al. [22] checked for the occurrence and duration of behavioral and electroencephalographic seizures, we performed detailed longitudinal analysis of multiple seizure parameters, such as number of spike trains, total spike train duration, average spike train duration, and total number of spikes. As it is known that gender and the estrous cycle in females in particular significantly affect seizure susceptibility/severity in rodents but also in humans [46–48], we performed for the first time an entire gender-specific seizure analysis of cortical M1 and hippocampal CA1 recordings (Figure 3).

The electroencephalographic seizures exhibit typical appearance with an initial spike followed by a complex spike train and subsequent postictal depression following seizure termination (Figures 1(a)(II), 1(b)(II), 1(c)(II), and 1(d)(II)). In contrast to other reports [21, 22, 26, 49], no APPswePS1dE9 mouse died during the time course of our experiments, particularly not from spontaneous prolonged status epilepticus as has been suggested previously [22]. In contrast to earlier studies that neglected a potential gender-specific interference, our results illustrate complex age- and sex-dependent seizure architecture in APPswePS1dE9 mice. There is an ongoing discussion on how neural degeneration can actually evolve in aberrant excitability in neural networks, such as the septohippocampal circuitry. There have been reports about loss of calbindin-positive immunoreactivity in dentate gyrus and abnormal NPY immunoreactivity in mossy fiber projections to CA3 [22, 26] with both alterations being interpreted as compensatory network changes. Studies by [50] further suggest that hyperactive neuronal clusters surrounding amyloid plaques in the cortex of APP/PS1 mice are involved in triggering seizures. In addition, it has been hypothesized that in the course of A*β* plaque formation disruption of voltage-gated ion channels [51–55] and plasma membrane potential destabilisation might result in seizure activity [26].

In addition to seizure analysis, we also had a detailed FFT based look at the cortical and hippocampal frequency characteristics, particularly the theta and gamma frequency range. Hippocampal theta rhythm is related to specific behavioral and cognitive processes including alertness, learning and memory, spatial navigation, and spatial memory. As the septohippocampal system is pathophysiologically affected in AD, one might expect an impairment in hippocampal rhythmicity, particularly in the theta frequency range [56–58]. Previous studies seemed to confirm this hypothesis showing that cognitive decline in AD is accompanied by decrease in theta activity [39, 56, 59], whereas others report an increase in theta activity [28, 56–58]. This paradoxical situation might be explainable given the sophisticated pathophysiology of the septohippocampal system and the heterogeneity, that is, dualistic theory of hippocampal theta activity, that is, hippocampal theta being differentiated into atropine-insensitive type I and atropine-sensitive type II theta [40, 60]. It has been reported that A*β* can modulate cholinergic and glutamatergic neurons in the septohippocampal system [61–64] and that the presence of A*β* in both the medial septum and the hippocampus can dampen theta rhythm in vitro and in vivo [62, 65–69] which is associated with cognitive impairment.

Scott et al. [59] reported about a decline in theta power at an age of 4 and 8 months in APPswePS1dE9 mice which was recorded under urethane anaesthesia and elicited by brainstem stimulation. Cayzac et al. [70] described alterations in local oscillatory activity in the theta range; however these findings were obtained under restrained tether recording conditions using mixed, unbalanced gender distribution in the study groups which is critical for interpretation.

Importantly, our study now clearly demonstrates under physiological, that is, spontaneous, conscious, and unrestrained, long-term recording conditions a reduction in theta power and a concomitant increase in gamma power that was not only age-dependent but also gender-specific (Figure 4). Whereas males exhibited a reduction in theta at an age of 14 wks that vanished while getting older, females exhibited theta power reduction at later stage of 18 and 19 wks of age. To our knowledge this is the first time that age- and gender-specific alterations in hippocampal theta and gamma architecture have been described in an AD mouse model. Importantly, an activity dependent analysis confirmed the antithetic theta-gamma power distribution particularly in females. These alterations were most prominent in the inactive state. It should be noted, that based on the dualistic theory, theta has to be differentiated into atropine-insensitive type I theta and atropine-sensitive type II theta. Active exploratory behavior, for example, is normally associated with type I theta, whereas type II theta is characteristic of alert immobility [26, 28, 67, 69, 71, 72]. Disruption of theta activity results in spatial memory deficits, whereas the restoration of theta-like rhythmicity restores learning capabilities in rats [73]. We conclude that inactive mice in both the dark and light phase predominately exhibit alert immobility which is accompanied by atropine-sensitive type II theta [74, 75]. Thus, our data suggest that female APPswePS1dE9 mice in particular demonstrate reduced atropine-sensitive type II theta at later stages which is likely to be due to septohippocampal impairment during AD development.

Previously, hippocampal in vitro preparations from 1-month-old TgCRND8 AD mice were shown to exhibit robust alterations in theta-gamma coupling although these mice expressed negligible amounts of A*β* [76]. In contrast to our study, Goutagny et al. [76] did not observe changes in theta or low gamma power in the TgCRND8 model of AD. Interestingly, Rubio et al. [41] described a decrease in both hippocampal theta and gamma power in hAPP AD mice which is antithetic to our results.

It is commonly accepted that, in the APPswePS1dE9 mouse model, A*β* burden of aged females appears to be more severe than in old males [23, 77–79]. However, examination of A*β* dynamics in this particular mouse line revealed additional age- and gender-related specificities. In 4-month-old APPswePS1dE9 mice, brain A*β* load has been reported to be dominated by A*β*_1–40_ rather than A*β*_1–42_ [23, 77]. Upon 6 months of age, A*β* ratio shifts towards A*β*_1–42_ that is maintained until end of life. Furthermore, a recent study showed that senile plaques in cortex and hippocampus can be detected in 3-month-old males but barely in females of the same age which means that A*β* plaques occur earlier in males [37]. This finding matches our observation that changes of theta and gamma power in CA1 region were firstly observed in males and later in females and stresses the tremendous importance of gender-specific analysis in AD mouse models. How exactly seizure incidence and power alterations of CA1 theta and gamma are linked with A*β* abundance, A*β*_1–42_/A*β*_1–40_ ratio, or initial occurrence of plaque formation has to be explored by future studies.

Recent studies by [80] in the cortex of APPswePS1dE9 mice suggest that there is a lack of GABAergic perisomatic synapses of basket cells on the surfaces of cortical pyramidal neurons that are in close contact with amyloid plaques. As perisomatic GABAergic synapses exhibit a dominant influence on the output behavior of pyramidal neurons, their structural impairment may result in hyperactivity of the neurons in close proximity to amyloid plaques. Gamma band rhythmogenesis is known to be inextricably tied to perisomatic inhibition, particularly via GABA A receptors [81]. As interneurons primarily determine the power of faster oscillations such as gamma [82–84], it seems unlikely that interneuronal pathology is solely responsible for the observed cortical gamma increase reported by [31, 85]. Generally, multiple mechanisms contribute to the generation of fast and slow oscillations [83], for example, cellular resonance properties [86, 87], recurrent excitation [88], excitation-inhibition interaction [89, 90], negative feedback [91], GABA receptor kinetics [83, 92–94], cellular bursting [95], or pacemaker activity [96, 97]. Thus, [31] hypothesized that multiple factors including basal forebrain projections and local hyperexcitability of cortical pyramidal cells based on increased resting membrane potential [22] may account for the observed alteration in cortical gamma power. Interestingly, [31] reported no changes in any hippocampal EEG range including gamma when recordings were done at 16-17 weeks of age. Importantly, the theta and gamma changes observed in our APPswePS1dE9 study predominantly occur at 18-19 weeks of age thus likely to be related to AD progression. Multiple hippocampal theta and gamma rhythm generators and underlying ionic mechanisms have been suggested [98] and further studies are necessary to unravel the detailed mechanisms underlying gender, age-specific, and activity-dependent alterations during AD development.

Our results demonstrate that hippocampal and cortical seizure and EEG power characteristics are related to both age and disease progression and clearly display gender specificity in the APPswePS1dE9 mouse model. These findings further point to the emerging role of novel gender-specific EEG fingerprints as potential early biomarkers of AD in the future.

## Supplementary Material

Supplementary Figure 1: Activity analysis in female controls and APPswePS1dE9 mice. Female animals from both groups were analyzed for motor activity (relative units) for different ages (14, 15, 18, 19 wks) and circadian periods (D1, L1, D2, L2, D1+D2, L1+L2). In females, no changes were observed in motor activity. Black, controls; gray, APPswePS1dE9.
Supplementary Figure 2: Electroencephalographic seizure analysis in controls and APPswePS1dE9. Both M1 (A) and CA1 (B) 48 hrs long-term EEG recordings were analyzed for electroencephalographic seizures using an automated seizure detection tool. Seizure parameters included the number of spike trains as well as the average spike train duration and were averaged for a single dark (D) or light (L) cycle for the various ages (14, 15, 18, 19 wks). Black, controls; gray, APPswePS1dE9.
Supplementary Figure 3: Gender specific frequency analysis in controls and APPswePS1dE9 mice during the active and inactive state. The mean EEG power [%] was calculated FFT based for males and females considering potential circadian rhythmicity (light / dark phase) and also the activity status (active, activity counts > 0; inactive, activity = 0). For CA1 deflections, theta (4-8 Hz), gamma (30-50 Hz) and gamma (50-70 Hz) were quantified. Frequency analysis was performed for all four ages (14, 15, 18, 19 wks). Black, controls; gray, APPswePS1dE9.

## Abbreviations

A*β*: Amyloid beta
AD: Alzheimer's disease
APP: Amyloid precursor protein
BACE: Beta-site amyloid precursor protein cleaving enzyme 1
CA: Cornu ammonis
CNS: Central nervous system
EEG: Electroencephalogram
ECoG: Electrocorticogram
FFT: Fast Fourier Transformation
i.p.: Intraperitoneal
PS: Presenilin.
