# Supplementary material for: Gender-Specific Hippocampal Dysrhythmia and Aberrant Hippocampal and Cortical Excitability in the APPswePS1dE9 Model of Alzheimer's Disease
Source: Neural Plast. 2016 Oct 20;2016:7167358. doi: 10.1155/2016/7167358 (PMC5093295; doi:10.1155/2016/7167358)
Supplement: Supplementary file 1 — Supplementary Figure 1: Activity analysis in female controls and APPswePS1dE9 mice. Female animals from both groups were analyzed for motor activity (relative units) for different ages (14, 15, 18, 19 wks) and circadian periods (D1, L1, D2, L2, D1+D2, L1+L2). In females, no changes were observed in motor activity. Black, controls; gray, APPswePS1dE9. Supplementary Figure 2: Electroencephalographic seizure analysis in controls and APPswePS1dE9. Both M1 (A) and CA1 (B) 48 hrs long-term EEG recordings were analyzed for electroencephalographic seizures using an automated seizure detection tool. Seizure parameters included the number of spike trains as well as the average spike train duration and were averaged for a single dark (D) or light (L) cycle for the various ages (14, 15, 18, 19 wks). Black, controls; gray, APPswePS1dE9. Supplementary Figure 3: Gender specific frequency analysis in controls and APPswePS1dE9 mice during the active and inactive state. The mean EEG power [%] was calculated FFT based for males and females considering potential circadian rhythmicity (light / dark phase) and also the activity status (active, activity counts > 0; inactive, activity = 0). For CA1 deflections, theta (4-8 Hz), gamma (30-50 Hz) and gamma (50-70 Hz) were quantified. Frequency analysis was performed for all four ages (14, 15, 18, 19 wks). Black, controls; gray, APPswePS1dE9. [file 7167358.f1.pdf]

Supplementary Figure 1 (Papazoglou et al., 2016)

Females

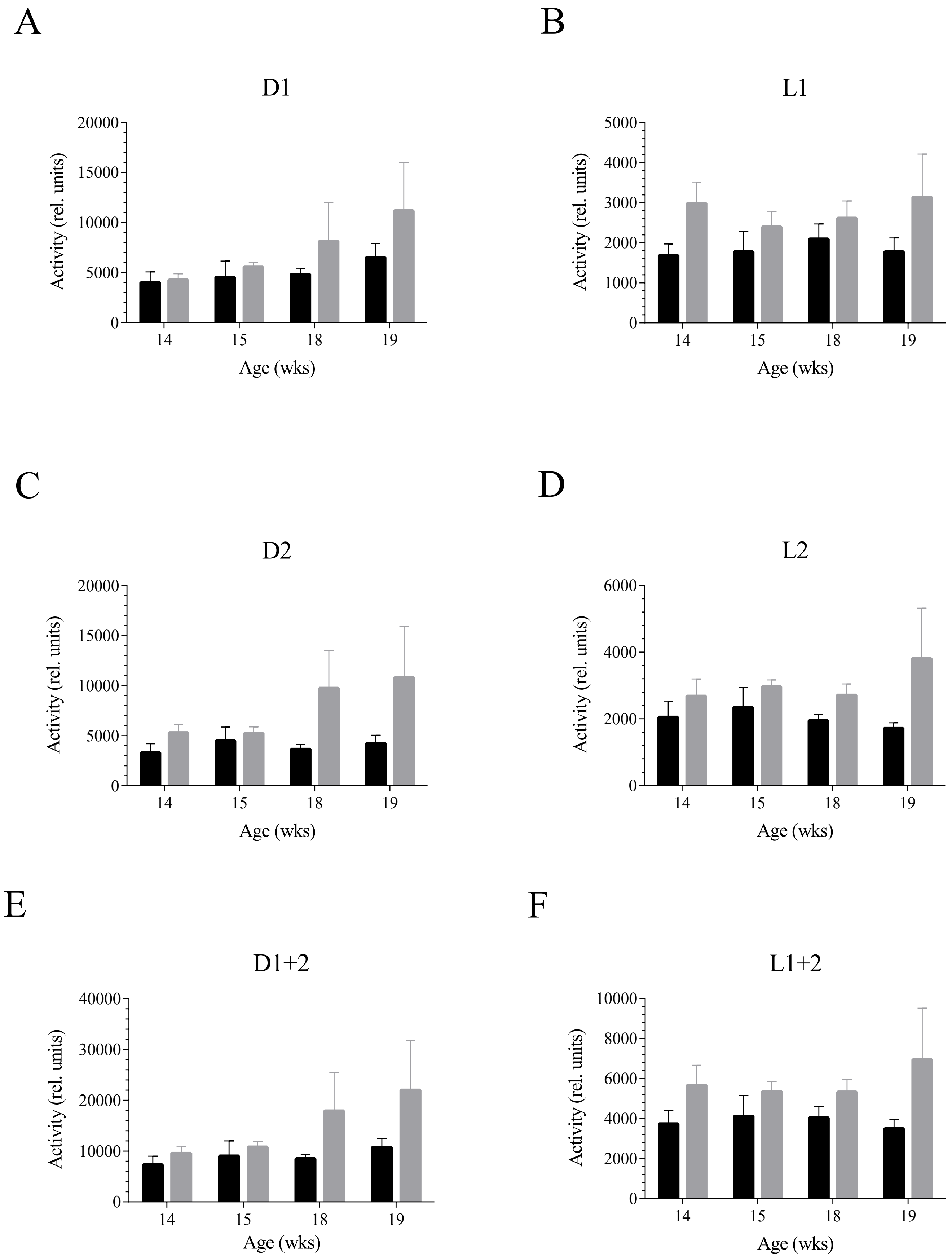

Supplementary Figure 2 (Papazoglou et al., 2016)

M1 deflection

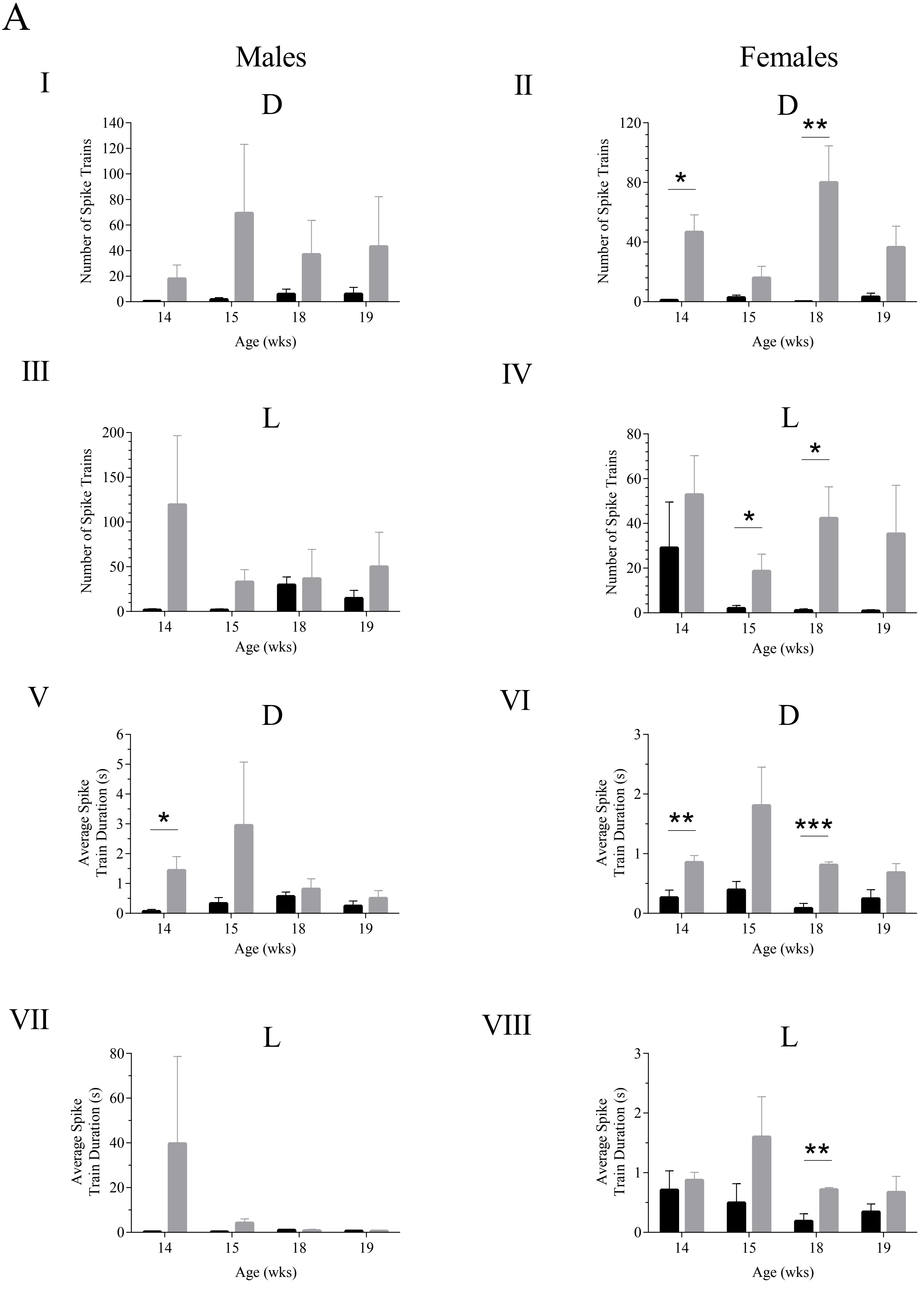

Supplementary Figure 2 (Papazoglou et al., 2016)

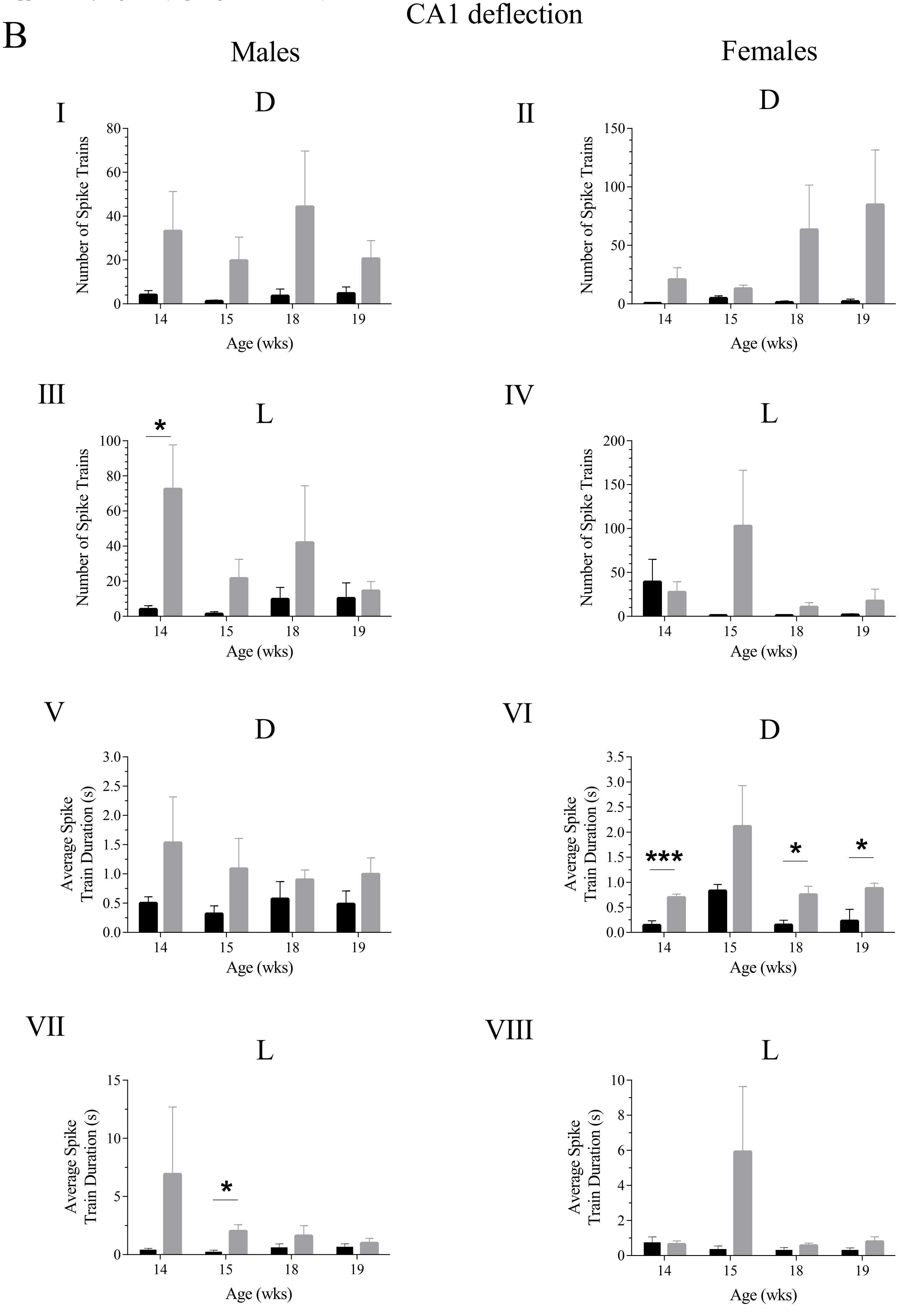

Supplementary Figure 3 (Papazoglou et al., 2016)

A

CA1 θ (4-8 Hz) - Dark cycle

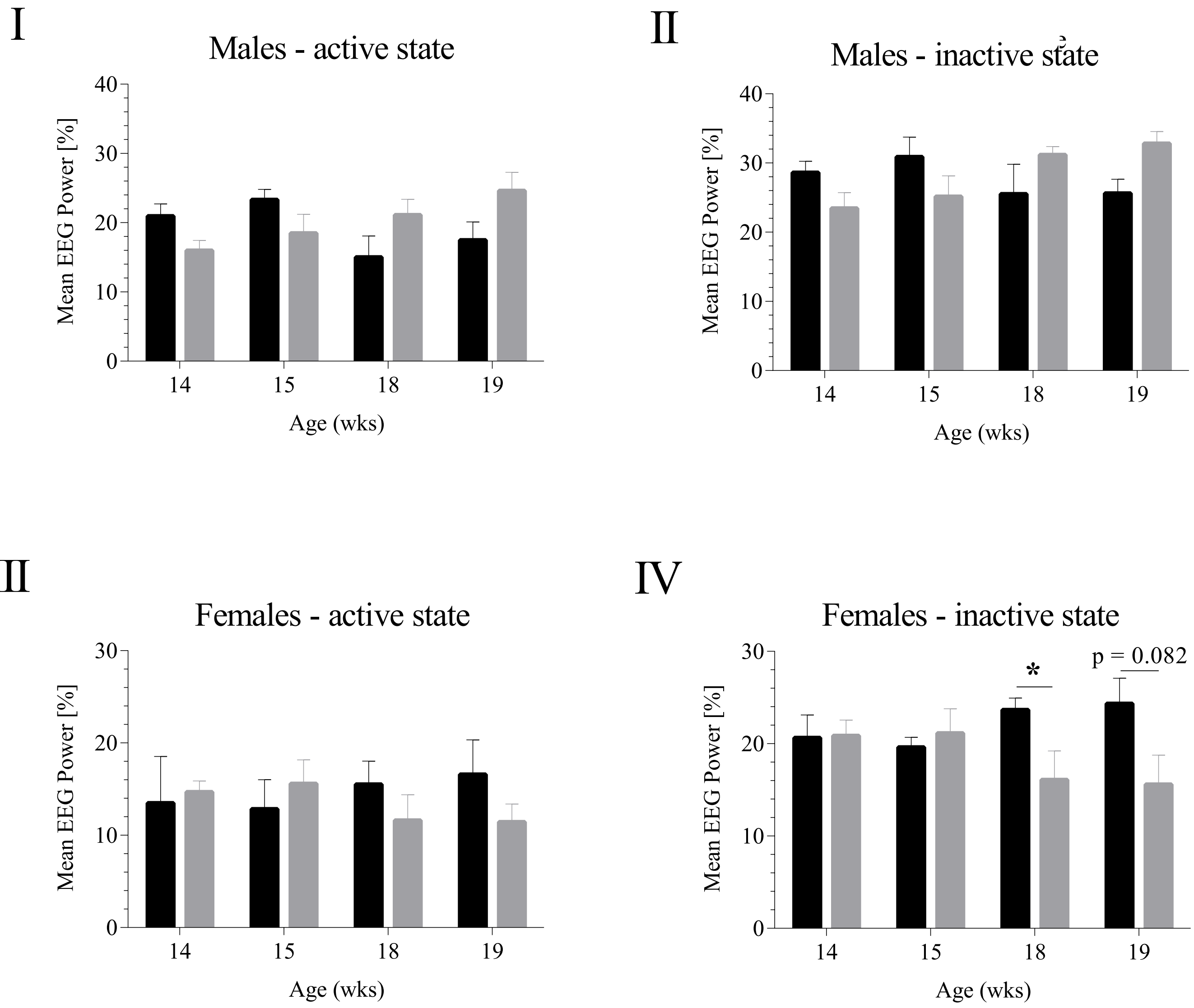

B

CA1 γ (30-50 Hz) - Dark cycle

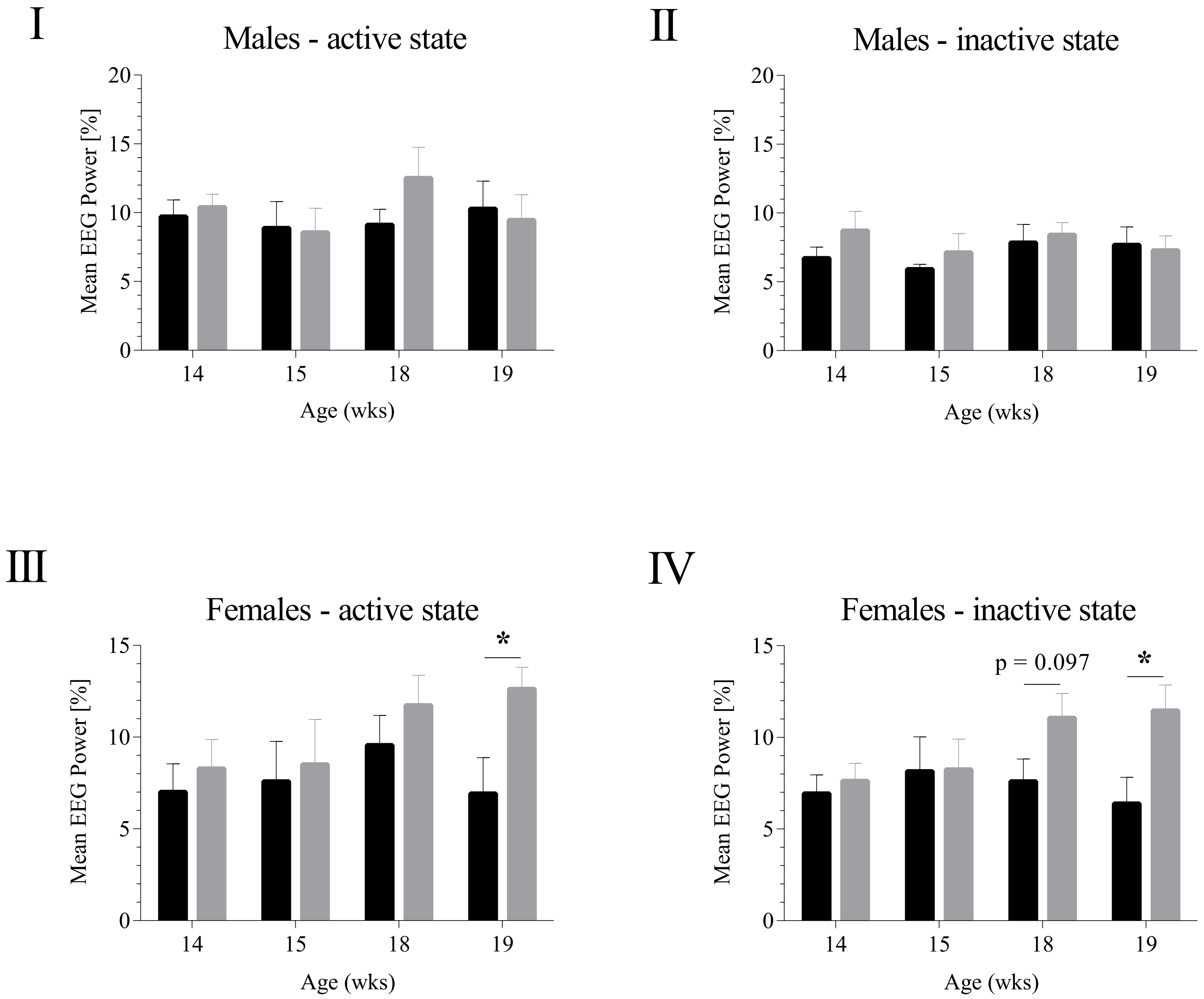

C

CA1 γ (50-70 Hz) - Dark cycle

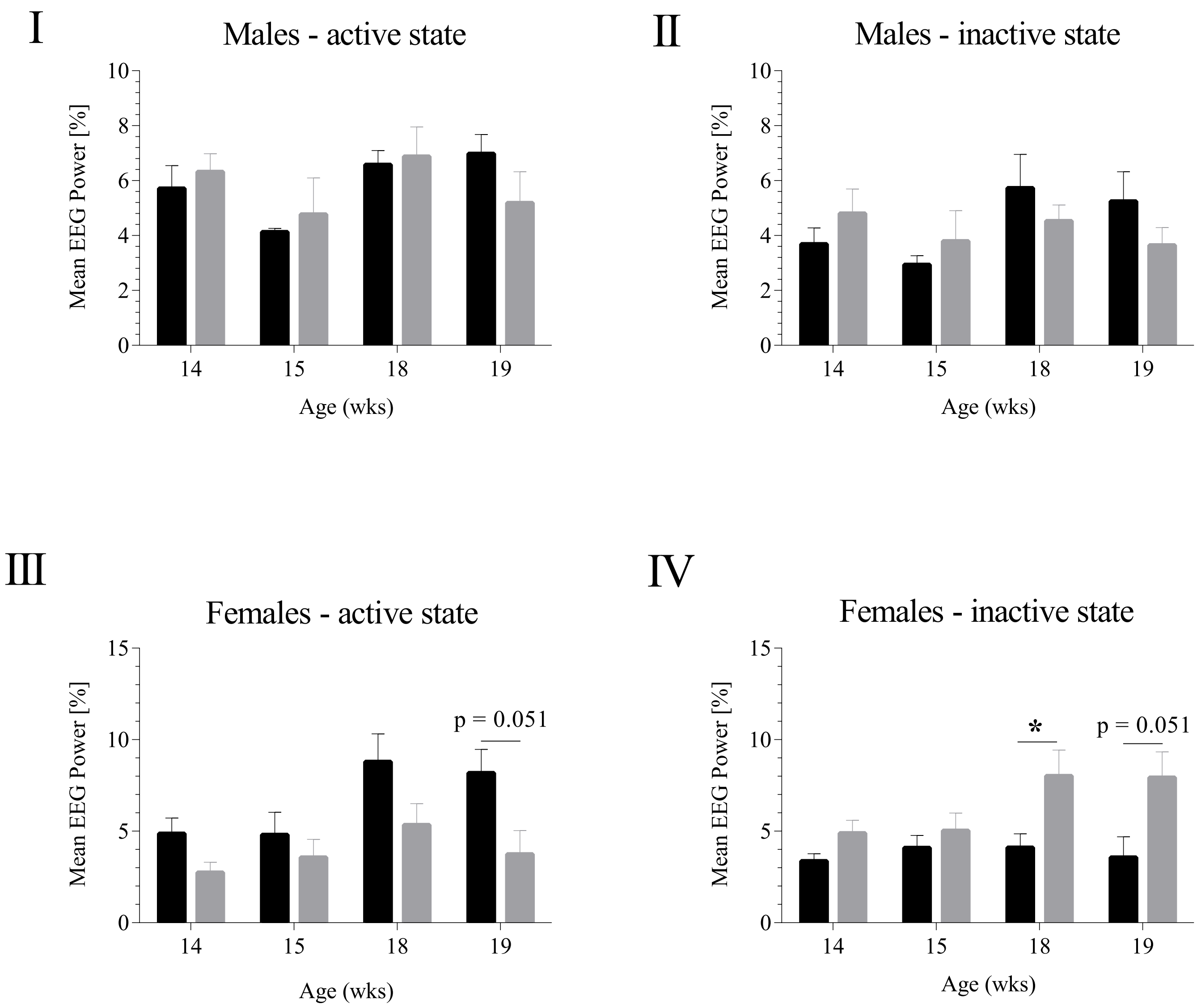

D

CA1 θ (4-8 Hz) - Light cycle

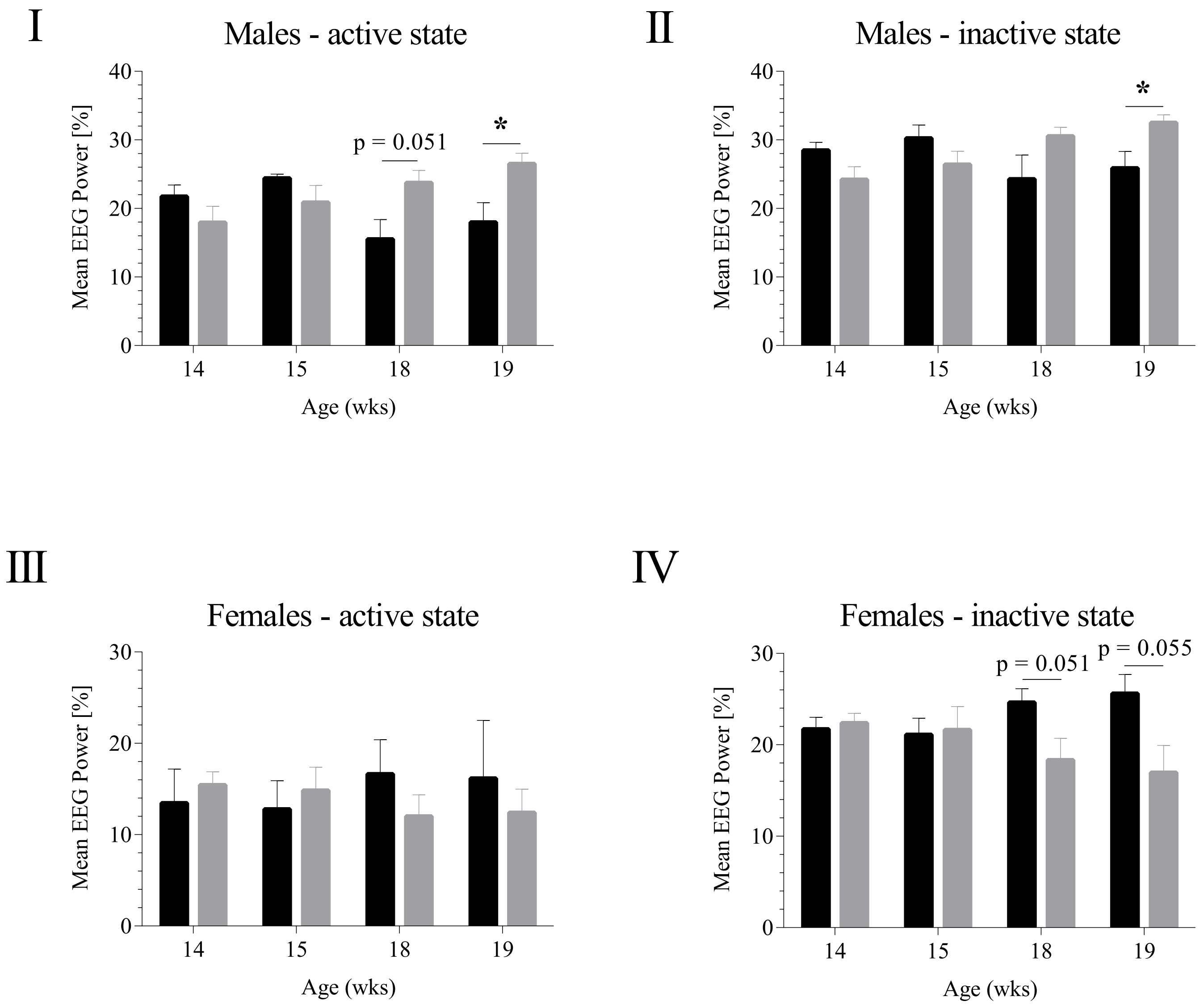

Supplementary Figure 3 (Papazoglou et al., 2016)

E

CA1 γ (30-50 Hz) - Light cycle

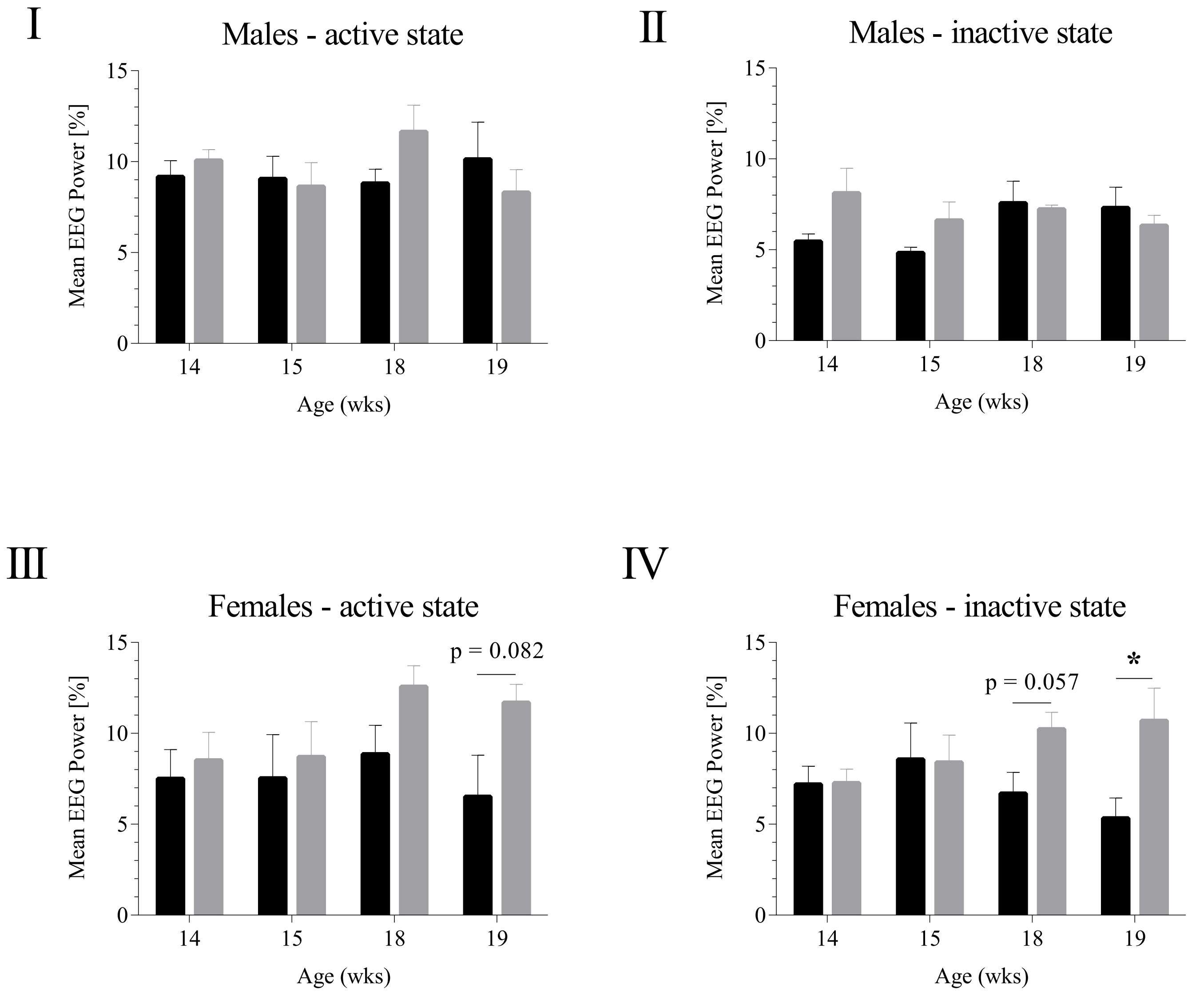

F

CA1 γ (50-70 Hz) - Light cycle

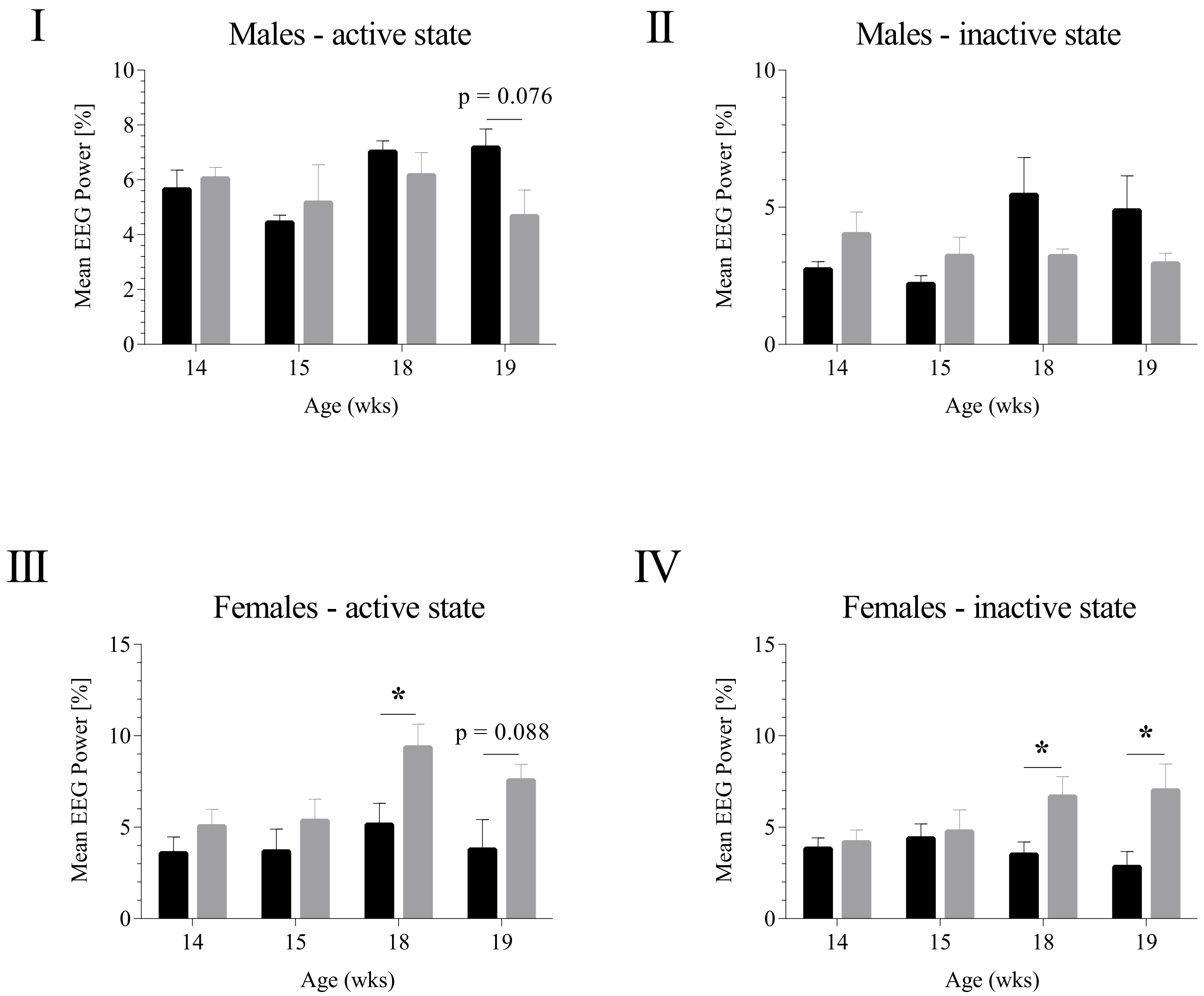

Supplementary Figure 1: Activity analysis in female controls and APPswePS1dE9 mice. Female animals from both groups were analyzed for motor activity (relative units) for different ages (14, 15, 18, 19 wks) and circadian periods (D1, L1, D2, L2, D1+D2, L1+L2). In females, no changes were observed in motor activity. Black, controls; gray, APPswePS1dE9.

Supplementary Figure 2: Electroencephalographic seizure analysis in controls and APPswePS1dE9. Both M1 (A) and CA1 (B) 48 hrs long-term EEG recordings were analyzed for electroencephalographic seizures using an automated seizure detection tool. Seizure parameters included the number of spike trains as well as the average spike train duration and were averaged for a single dark (D) or light (L) cycle for the various ages (14, 15, 18, 19 wks). Black, controls; gray, APPswePS1dE9.

Supplementary Figure 3: Gender specific frequency analysis in controls and APPswePS1dE9 mice during the active and inactive state. The mean EEG power [%] was calculated FFT based for males and females considering potential circadian rhythmicity (light / dark phase) and also the activity status (active, activity counts > 0; inactive, activity = 0). For CA1 deflections, theta (4-8 Hz), gamma (30-50 Hz) and gamma (50-70 Hz) were quantified. Frequency analysis was performed for all four ages (14, 15, 18, 19 wks). Black, controls; gray, APPswePS1dE9.
